# Inhalation of Salvianolic Acid B Prevents Fine Particulate Matter-Induced Acute Airway Inflammation and Oxidative Stress by Downregulating the LTR4/MyD88/NLRP3 Pathway

**DOI:** 10.1155/2022/5044356

**Published:** 2022-06-27

**Authors:** Yan Guan, Liucheng Li, Liandi Kan, Qiangmin Xie

**Affiliations:** ^1^Affiliated Sir Run Run Shaw Hospital, Zhejiang University School of Medicine, Hangzhou 310020, China; ^2^Zhejiang Respiratory Drugs Research Laboratory of State Food and Drug Administration of China, Zhejiang University School of Medicine, Hangzhou 310058, China; ^3^The Children's Hospital, Zhejiang University School of Medicine, National Clinical Research Center for Child Health, Hangzhou 310052, China

## Abstract

Air pollution is a serious threat to human health. Inhaled fine particulate matter (PM2.5) can cause inflammation and oxidative stress in the airway; however, the mechanisms responsible for this effect have yet to be elucidated and there are no specific drugs that can prevent and treat this condition. In the present study, we investigated the effects and mechanisms underlying the inhalation of salvianolic acid B (SalB) on PM2.5-induced airway inflammation and oxidative stress. We used a PM2.5-induced mouse model of airway inflammation and oxidative stress, along with a human epithelial cell model, to study the action and mechanisms of SalB by histopathology, real-time PCR, enzyme-linked immunosorbent assays, flow cytometry, and western blotting. SalB treatment markedly inhibited the PM2.5-induced increase in the number of neutrophils and macrophages in bronchoalveolar lavage fluid, improved the infiltration of inflammatory cells in lung tissue, and reduced injury in the alveolar septum. Furthermore, SalB reduced the mRNA and protein levels of interleukin- (IL-) 1*β*, tumor necrosis factor- (TNF-) *α*, keratinocyte (KC), and transforming growth factor- (TGF-) *β*1 in lung tissues and the protein levels of IL-1*β*, TNF-*α*, IL-8, IL-6, and TGF-*β*1 in human epithelial cells. SalB treatment also significantly prevented the reduction of levels of superoxide dismutase, catalase, glutathione, and glutathione peroxidase in lung tissue and reduced the levels of reactive oxygen species in human epithelial cells induced by PM2.5. Furthermore, SalB and the myeloid differentiation primary response 88 (MyD88) inhibitor ST2825 inhibited the expression levels of toll-like receptor 4 (TLR4), MyD88, tumor necrosis factor receptor associated factor 6 (TRAF-6), and NOD-like receptor protein 3 (NLRP3), as well as the phosphorylation of downstream Erk1/2 and P38 in lung tissue and epithelial cells. SalB protects against PM2.5-induced airway inflammation and oxidative stress in a manner that is associated with the inhibition of the TLR4/MyD88/TRAF-6/NLRP3 pathway and downstream signals ERK1/2 and P38.

## 1. Introduction

Fine particulate matter (PM2.5) refers to suspended solids that are less than or equal to 2.5 *μ*m in size and can contain a range of complex elements, including organic carbons, ions, heavy metals, bacteria, and viruses. PM2.5 can readily gain access to the alveolar terminals, pass through the alveoli into the blood, and then be transported to other organs and tissues [1, 2]. The inhalation of PM2.5 into the respiratory tract leads to the stimulation of epithelial cells in the airways; this releases inflammatory factors that induce the accumulation of inflammatory cells in the lung tissue and an oxidative stress response, ultimately leading to acute lung inflammation and oxidative stress and inducing asthma attacks or exacerbating asthma severity [3, 4]. Previous studies have confirmed that the airway inflammation and oxidative stress induced by PM2.5 are related to the NOD-like receptor protein 3 (NLRP3) inflammasome. When activated, the NLRP3 inflammasome stimulates activation of caspase-1 and the subsequent conversion of prointerleukin-1*β* into its mature bioactive form (IL-1*β*). IL-1*β* is a multifunctional proinflammatory factor that plays a key role in the process of PM2.5-induced airway inflammation [5, 6]. In addition, there are other pathways by which PM2.5 can promote inflammation. For example, PM2.5 exposure decreased miR-331 expression via the ROS/PI3K/Akt pathway, resulting in an increase in the IKK-*β* expression and sustained NF*κ*B activation in human airway epithelial cells [7]. PM2.5 was shown to promote the transformation of macrophages into foam cells in the RAW264.7 cell line by upregulating the expression of the TLR4/MyD88/NF*κ*B pathway [8]. Furthermore, both long- and short-term exposures to PM2.5 have been shown to increase the development of atherosclerosis in ApoE ^−/−^ mice [8] However, no specific drug has been developed for PM2.5. Consequently, there is an urgent need to develop potential targets and drugs for the prevention and treatment of PM2.5.

The natural compounds or traditional Chinese medicine (TCM), as a human aliment or nutritional supplements or therapeutic medicines, is widely used to against inflammation, modify the microenvironment, and regulate the function of immune cells in the various inflammatory and immune diseases including tumor immune escape [9]. Salvianolic acid B (SalB) is one of the main water-soluble active compounds in *Salvia miltiorrhiza*. *Salvia miltiorrhiza* root (Danshen) is known for its anti-inflammatory, antioxidation, and antifibrosis effects and has been used for a long period of time to treat a variety of diseases, including those affecting the cardiovascular system, the brain, lung, liver, kidney, and also a range of cancers [10–12]. In our previous research, we used the ovalbumin-induced mouse model of asthma to demonstrate that SalB attenuates hyperresponsiveness in the airway, infiltration by eosinophils, and the hyperplasia of goblet cells and that these effects were regulated by MUC5AC protein in response to the inhibition of ERK1/2/P38 signaling [13]. However, it is not yet known whether SalB can alleviate PM2.5-induced airway inflammation. Existing pharmacokinetic studies have shown that the absolute bioavailability of SalB when administered orally is comparatively low [14, 15]. Therefore, we considered the potential effect of SalB when inhaled as an aerosol. Delivery by inhalation provides an excellent opportunity for local treatment in the lungs. Ideally, this form of local administration results in a high concentration of the drug at the target organ and high levels of efficacy. However, the systemic concentration of the inhaled drug remains at low levels; therefore, adverse reactions are rare. In the present study, we evaluated the effect of SalB inhalation on the prevention and treatment of PM2.5-induced airway inflammation and oxidative stress in a mouse model. We also performed a range of *in vitro* experiments to investigate PM2.5-induced oxidative stress and inflammatory reaction in human bronchial epithelial cells and explore the potential signaling pathways involved.

## 2. Materials and Methods

### 2.1. Reagents and Antibodies

Fine particulate matter (PM2.5) standard was purchased from Wuhan Zhuoyan Huabiao Technology Co., Ltd. (Wuhan, China). The PM2.5 standard was autoclaved to exclude bacteria and viruses. According to chemical analysis, the major elements contained in the PM2.5 standard were as follows: 1.5 *μ*g/mg of Pb, 3.9 *μ*g/mg of Al, 4.1 *μ*g/mg of Ca, 5.1 *μ*g/mg of Na, 5.5 *μ*g/mg of Zn, 6.3 *μ*g/mg of Fe, 11 *μ*g/mg of Si, and 14 *μ*g/mg of K. The standard also contained 79 *μ*g/mg of organic carbon and 165 *μ*g/mg of elemental carbon.

Salvianolic acid B (SalB) was obtained from Shanghai PureOne Bio Technology Co. Ltd. (96% purity, Shanghai, China) as a white powder and in a water-soluble form. SalB was prepared with saline as a range of solutions for inhalation (2.5, 7.5, and 15.0 mg/mL). The SalB solution was sprayed into the inhalation exposure tower with a jet nebulizer (BARI Co. Ltd., Germany). After the aerosol containing SalB had been filled, we removed 48 mL of aerosol from the exposure tower with a 50-mL syringe (containing 2 mL of HPLC mobile phase). The syringe was sealed with a rubber stopper and allowed to stand for 1 min, and the contents of the syringe were shaken violently and allowed to stand for 1 min. Samples were taken in duplicate, and a total of 96 mL of aerosol was collected for analysis. Once collected, the concentration of drugs in each of the 2 mL samples was detected by reversed-phase high-performance liquid chromatography (Shimadzu, Japan) using a C_18_ column (150 × 4.6 mm, 5 *μ*m) with acetonitrile and trifluoroacetic acid as the mobile phase and diluent, respectively. The flow rate was 2 mL/min, and the injection volume was 20 *μ*L under 225 nm ultraviolet spectrophotometry. Finally, the administered dose of SalB (2.5, 7.5, and 15 mg/mL) was converted by a formula to the actual inhaled doses of approximately 0.3, 0.9, and 1.8 mg/kg, respectively; these data referred to mice in the exposure tower inhaling the medicine for a total of 8 min. In addition, before the study, we also compared the drug concentration in the lung tissue and plasma after SalB inhalation versus gavage administration to mice (Supplementary Materials, Table S1 and Figures S1 and S2a-f).

Commercial kits were purchased from Jiancheng Bioengineering Institute (Nanjing, China) in order to determine the levels of superoxide dismutase (SOD), catalase (CAT), glutathione (GSH), and glutathione peroxidase (GSH-Px). 2′, 7′-Dichlorofluorescein diacetate (DCFH-DA) was purchased from AAT Bioquest (Sunnyvale, CA). PCR primers for mouse IL-1*β*, TNF-*α*, KC, TGF-*β*1, toll-like receptor 4 (TLR4), myeloid differentiation primary response 88 (MyD88), TNF receptor associated factor 6 (TRAF-6), and NLRP3 were all purchased from Shanghai Bioengineering (Shanghai, China). Enzyme-linked immunoassay (ELISA) kits for mouse IL-1*β*, TNF-*α*, KC, and TGF-*β*1 and human IL-1*β*, TNF-*α*, IL-8, IL-6, and TGF-*β*1 were purchased from USCN Life Science, Inc. (Wuhan, China). Fetal bovine serum (FBS) was purchased from Hangzhou Sijiqing Biological Engineering Material Co., Ltd. (Hangzhou, China). RPMI 1640 medium, streptomycin, and penicillin were purchased from HyClone (Utah, USA). Primary antibodies for GAPDH, TLR4, MyD88, TRAF-6, NLRP3, extracellular signal-regulated kinase 1/2 (Erk1/2), and P38 mitogen-activated protein kinase (P38) were obtained from Cell Signaling Technology (Danvers, MA, USA). Secondary antibodies, electrophoresis apparatus, and electrophoresis chambers were obtained from Bio-Rad (Hercules, CA, USA).

### 2.2. Animals

Male ICR mice (22 ± 2 g, 8 weeks old) were purchased from the Experimental Animal Center at the Zhejiang Academy of Medical Sciences (Hangzhou, China). The animals were housed at the Laboratory Animal Center of Zhejiang University in isolated ventilated cages (4-5 mice/cage) under a 12 h light/12 -h dark cycle and received food and water *ad libitum*. The Institutional Animal Care and Use Committee of Zhejiang University approved the experimental protocols used in this study. To investigate the acute effect of PM2.5 in the development of airway inflammation and oxidative stress, the mice were randomly assigned to six groups: a control (saline) group, a model group (PM2.5), a SalB (2.5 mg/mL, equivalent to 0.3 mg/kg)+PM2.5 group, a SalB (7.5 mg/mL, equivalent to 0.9 mg/kg)+PM2.5 group, a SalB (15 mg/mL, equivalent to 1.8 mg/kg)+PM2.5 group, and a ST2825 (5 mg/mL, equivalent to 0.6 mg/kg) group. Mice in the PM2.5 groups were initially treated with 10 *μ*g of PM2.5 (10 *μ*L) *via* a tracheal spray needle and intratracheal instillation; subsequently, PM2.5 was administered intranasally on a daily basis for the next 5 days. Mice in the control group were treated with 10 *μ*L of normal saline. The SalB was sprayed into the exposure tower with a jet nebulizer (BARI Co. Ltd., Germany) for 8 min/day for 30 min prior to PM2.5 treatment.

### 2.3. Bronchoalveolar Lavage

Six days after exposure to PM2.5, mice were anesthetized by the inhalation of isoflurane. Samples of bronchoalveolar lavage fluid (BALF) were then collected by cannulating the trachea and performing lavage with phosphate buffer solution (PBS) containing 1% bovine serum albumin (BSA) and 5000 IU/L heparin. The total leukocytes, neutrophils, macrophages, and lymphocytes were counted in BALF after centrifugation, smear, and staining.

### 2.4. Histopathological and Immunohistochemical Examination

Samples of tissue from the left lung of each experimental animal were fixed in 10% formalin for 15 days. The samples were sectioned into 3–4 *μ*m thick sections. The sections of lung tissue were then stained with hematoxylin-eosin (H&E) to evaluate the severity of inflammatory cell infiltration (neutrophils and macrophages) and edema in the alveolar septum. A scoring system was then used to assess severity (score) of inflammatory damage, as described previously [13]. Immunohistochemical (IHC) analysis of MyD88 was performed in accordance with the protocol provided with the Cell Signaling Technology Complex Kit (Danvers, MA, USA). Image Pro version 6.1 software (Houston, USA) was used for quantitative analysis of the staining. All analyses were performed in a blinded manner.

### 2.5. Cell Culture

Human bronchial epithelial (16HBE) cells were acquired from the Cell Bank of the Chinese Academy of Sciences (Shanghai). The cells were continuously cultured in RPMI 1640 medium supplemented with 10% fetal bovine serum, 100 U/mL of penicillin, and 100 ng/mL of streptomycin at 37°C under a humidified atmosphere of 5% CO_2_/95% air.

### 2.6. Determination of Oxidative Stress

The 16HBE cells were treated with SalB and ST2825 and then exposed to PM2.5 (200 *μ*g/mL) for 24 h. Next, cells were collected by centrifugation and resuspended in 2′,7′-dichlorodihydrofluorescein diacetate (DCFH-DA) solution (5 *μ*M/L) at 37°C for 15 min. The cell suspensions were mixed by inverting every 3-5 min to fully expose cells to the probe. The cells were then washed three times with cell culture medium to remove the DCFH-DA that had not entered the cells. Finally, the fluorescence intensity of the cell reactive oxygen species (ROS) was detected by flow cytometry (Beckman Coulter, USA).

Supernatants were extracted from lung tissue homogenates and the culture media of 16HBE cells. Then, we determined the levels of SOD, CAT, GSH, and GSH-Px, in the lung tissues, by using a range of commercial kits in accordance with the manufacturer's instructions (Jiancheng Bioengineering Institute, Nanjing, China).

### 2.7. RT-PCR

First, we used a TRIzol kit (Takara Bio, Dalian, China) to extract total RNA from lung tissues and cells. Next, we used an ABI 7500 PCR system (Applied Biosystems) to determine mRNA expression levels in cells and tissues. Quantification of mRNA expression in cells and lung tissue samples was performed using UltraSYBR Mixture (Takara Bio, Dalian, China). The conventional procedure was used for PCR cycling. GAPDH was used as an internal control, and mRNA levels were calculated using the ^2-*ΔΔ*^Ct method (relative) [16]. All primers were tested using a basic local alignment search tool to determine their selectivity. The primer sequences are shown in Table 1.

### 2.8. Western Blotting

Total proteins were extracted from cells and lung tissues using radioimmunoprecipitation (RIPA) lysis buffer kits (Boster Co. Ltd., CA). Protein extracts were then separated by 10% sodium dodecyl sulfate-polyacrylamide gel electrophoresis (SDS-PAGE) and transferred onto polyvinylidene fluoride (PVDF) membranes. Then, we used rapid blocking buffer (Beyotime, China) to block the membranes at room temperature for 15 min and then incubated the membranes overnight at 4°C with an appropriate concentration of each primary antibody (1 : 200–1000, anti-TLR4, anti-MyD88, anti-TRAF-6, anti-NLRP3, anti-Erk1/2, anti-P38, and anti-GAPDH), respectively. The next morning, membranes were washed and incubated with secondary antibody (Cell Signaling Technology, Beverly, MA, USA) for 1 h. Then, band densities were quantified using ImageJ software (version 1.38e, NIH, Bethesda, MD) and normalized to their respective loading controls.

### 2.9. Statistical Analysis

All statistical analysis was carried out with SPSS version 20.0 (SPSS Inc., Chicago, IL, USA) and GraphPad Prism 6.0 (GraphPad Software, La Jolla, CA, USA). Differences between the mean values of multiple groups were analyzed by one-way analysis of variance (ANOVA) followed by the Student-Newman-Keuls test. *P* < 0.05 was statistically significant.

## 3. Results

### 3.1. PM2.5-Induced Lung Inflammation and MyD88 Expression In Vivo

**Figure 1:**
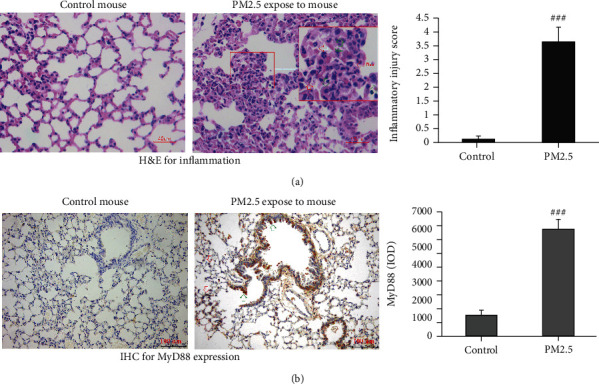
PM2.5 induced lung inflammation and upregulated MyD88 *in vivo*. (a) A representative image of lung inflammation (the infiltration of numerous neutrophils and macrophages) in lung tissues stained with hematoxylin-eosin. (b) The expression of MyD88; lung tissues were stained by immunohistochemistry. Data are expressed as mean ± S.E.M.; *n* = 6/group. ^###^*P* < 0.001 compared with the control group.

First, we first investigated the effects of PM2.5 exposure on airway inflammation and the expression of MyD88 in lung tissues. As shown in Figure 1, after 6 days of exposure to PM2.5, the lung tissue of experimental mice showed clear signs of serious damage, including infiltration by a large number of neutrophils and macrophages and edema of the alveolar septum (Figure 1(a)). In addition, MyD88 protein was strongly expressed in epithelial cells at the medial trachea and in regions of lung tissue showing the infiltration of macrophages in the lung tissues (Figure 1(b)).

### 3.2. SalB Relieved PM2.5-Induced Lung Inflammation In Vivo

**Figure 2:**
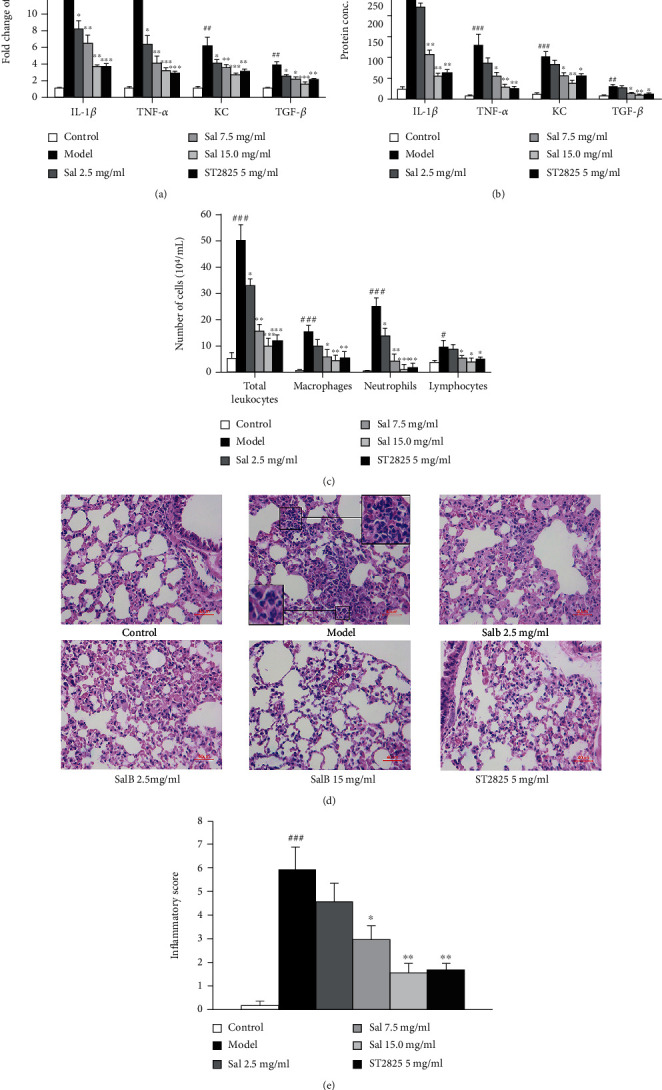
The effects of SalB on PM2.5-induced lung inflammation *in vivo*. (a, b) Lung tissue homogenates were prepared for mRNA and protein assays. The expression levels of IL-1*β*, TNF-*α*, KC, and TGF-*β*1 mRNA and protein were measured by Q-PCR and ELISA. (c) The number of total leukocytes, neutrophils, macrophages, and lymphocytes in BALF. (d) Representative images of inflammatory cells infiltrating into lung tissues, as demonstrated by H&E staining. (e) Semiquantitative score analysis for the infiltration of pulmonary inflammatory cells and injury. Data are expressed as mean ± S.E.M.; *n* = 10/group of two independent experiments. ^#^*P* < 0.05, ^##^*P* < 0.01, and ^###^*P* < 0.001 compared with the control group; ^∗^*P* < 0.05, ^∗∗^*P* < 0.01, and ^∗∗∗^*P* < 0.001 compared with the model group (exposure to PM2.5).

Analysis showed that exposure to PM2.5 induced a significant increase in the mRNA and protein expression levels of IL-1*β*, TNF-*α*, KC, and TGF-*β*1 in lung tissues; these were reduced by treatment with SalB (2.5, 7.5, and 15 mg/mL) in a dose-dependent manner or by treatment with ST2825, an MyD88 inhibitor (5 mg/mL), compared with the model group (Figures 2(a) and 2(b)). In addition, there was a significant reduction in the concentrations of total leukocytes, macrophages, and neutrophils, in mice that inhaled SalB or ST2825 when compared with the model group (Figure 2(c)). Pathological analysis showed that the inhalation of SalB or ST2825 alleviated alveolar septal edema and the influx of neutrophils and macrophages into the alveolar spaces (Figures 2(d) and 2(e)). These observations provide preliminary evidence for SalB in the inhibition of acute airway inflammation induced by PM2.5.

### 3.3. SalB Alleviated PM2.5-Induced Oxidative Stress In Vivo

**Figure 3:**
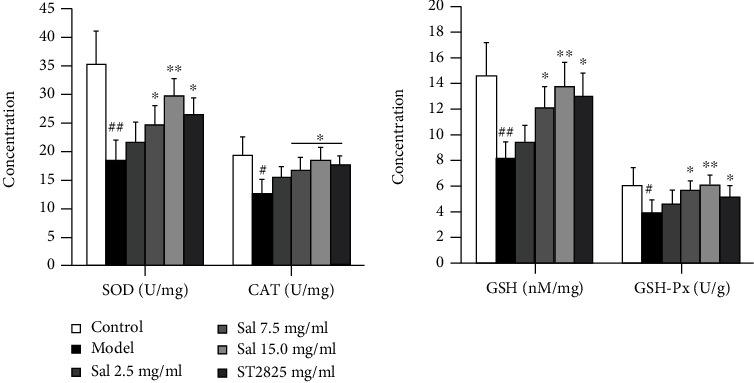
Effects of SalB on PM2.5-induced oxidative stress *in vivo*. Lung tissues were harvested 24 h after the final exposure to PM2.5. We detected the levels of SOD, CAT, GSH, and GSH-Px in the supernatant of lung tissue homogenates. Data are expressed as mean ± S.E.M.; *n* = 10/group of two independent experiments. ^#^*P* < 0.05 and ^##^*P* < 0.01 compared with the control group, ^∗^*P* < 0.05 and ^∗∗^*P* < 0.01 compared with the model group.

Results showed that PM2.5 exposure induced a significant reduction in SOD, CAT, GSH, and GSH-Px levels in mouse lung tissues; these changes were alleviated, in a dose-dependent manner, by treatment with SalB (2.5, 7.5, and 15 mg/mL) or ST2825 (5 mg/mL) (Figure 3). These observations revealed that SalB inhibited PM2.5-induced acute oxidative stress.

### 3.4. SalB Inhibited the PM2.5-Induced Expression of TLR4, MyD88, TRAF-6, and NLRP3 In Vivo

**Figure 4:**
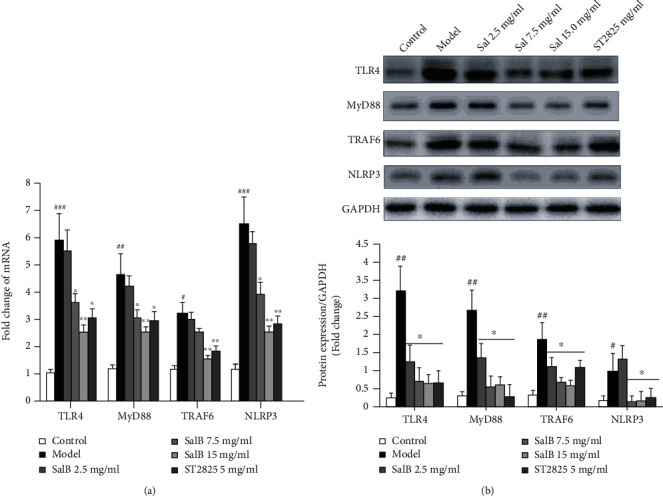
Effects of SalB on the PM2.5-induced expression of TLR4, MyD88, TRAF-6, and NLRP3 mRNA and protein *in vivo*. (a) The mRNA expression levels of *TLR4*, *MyD88*, *TRAF-6*, and *NLRP3* were detected by RT-PCR (*n* = 6 per group). Data represent means ± S.E.M. from three independent experiments. (b) The expression levels of TLR4, MyD88, TRAF-6, and NLRP3 protein were assessed by western blotting (*n* = 3/group). Data represent mean ± S.E.M. from three independent experiments. ^#^*P* < 0.05, ^##^*P* < 0.01, and ^###^*P* < 0.001 compared with the control group; ^∗^*P* < 0.05 and ^∗∗^*P* < 0.01 compared with the model group.

PM2.5 induced a significant increase in the mRNA and protein expression of TLR4, MyD88, TRAF-6, and NLRP3 in lung tissues; these expression levels were reduced, in a dose-dependent manner, by treatment with SalB (2.5, 7.5, and 15 mg/mL) or ST2825 (5 mg/mL) (Figures 4(a) and 4(b)). These observations revealed that SalB inhibited PM2.5-induced acute airway inflammation and oxidative stress *via* the TLR4/MyD88/TRAF-6/NLRP pathway.

### 3.5. SalB Reduced the PM2.5-Induced Expression of Inflammatory Cytokines and Oxidative Stress In Vitro

**Figure 5:**
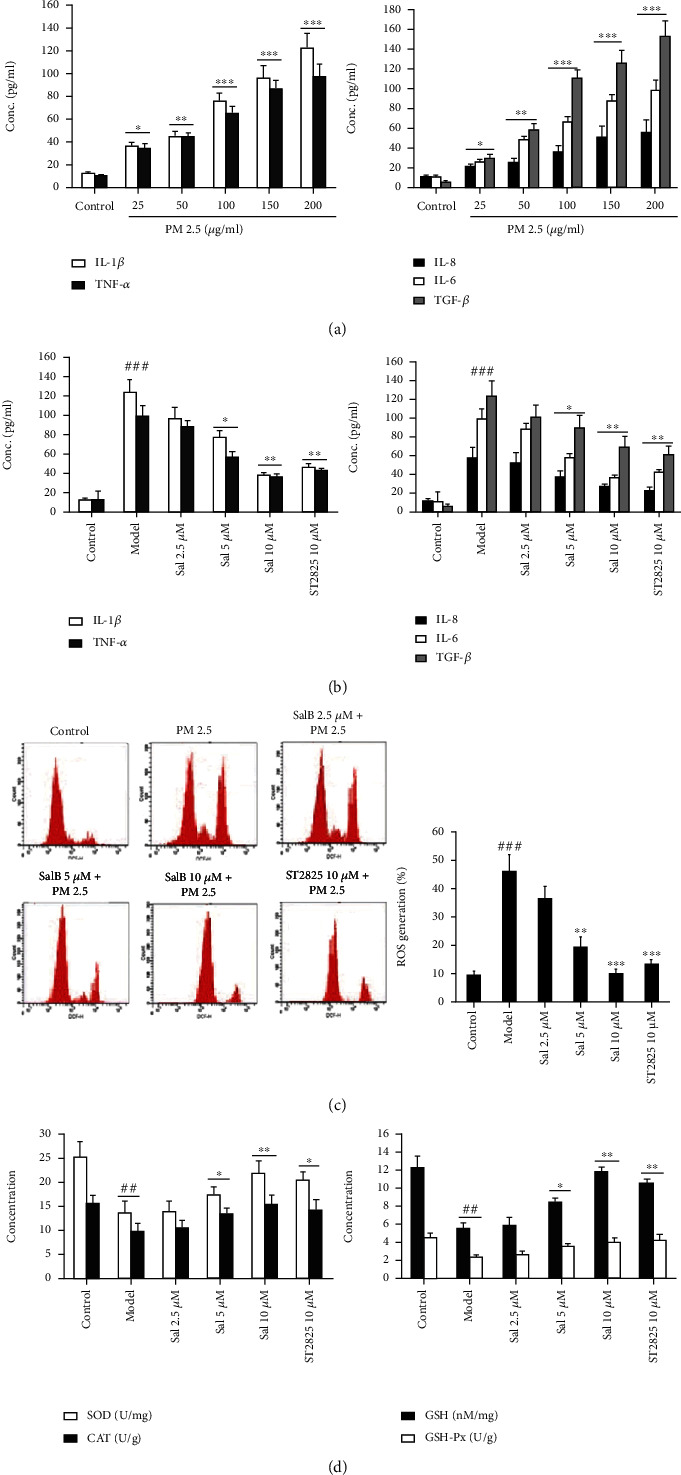
Effects of SalB on PM2.5-induced inflammatory cytokines and oxidative stress *in vitro*. (a) Human bronchial epithelial (16HBE) cells were exposed to PM2.5 at various concentrations (25-200 *μ*g/mL) for 24 h. PM2.5 caused a dose-dependent elevation in the levels of IL-1*β*, TNF-*α*, IL-8, IL-6, and TGF-*β*1 proteins. (b) Treatment with SalB resulted in a dose-dependent reduction in IL-1*β*, TNF-*α*, IL-8, IL-6, and TGF-*β*1 protein. (c) Treatment with SalB resulted in a dose-dependent reduction in ROS. (d) Treatment with SalB resulted in a dose-dependent increase in the levels of SOD, CAT, GSH, and GSH-Px. Data are expressed as mean ± S.E.M.; *n* = 6/group of three independent experiments. ^#^*P* < 0.05, ^##^*P* < 0.01, and ^###^*P* < 0.001 compared with the control group; ^∗^*P* < 0.05 and ^∗∗^*P* < 0.01 compared with the model group.

To verify the role of SalB *in vivo*, we next investigated the effect of SalB on PM2.5-induced inflammation and oxidative stress in human bronchial epithelial cells. Exposure to PM2.5 at various concentrations (25–200 *μ*g/mL) for 24 h caused a dose-dependent elevation in the levels of IL-1*β*, TNF-*α*, IL-8, IL-6, and TGF-*β*1 protein (Figure 5(a)). As shown in Figure 5(b), thet reatment of 2.5, 5, and 10 *μ*M of SalB resulted in a dose-dependent reduction in the protein levels of IL-1*β*, TNF-*α*, IL-8, IL-6, and TGF-*β*1. In addition, there was a dose-dependent reduction in ROS levels and an increase in the levels of SOD, CAT, GSH, and GSH-Px following pretreatment with SalB or ST2825 (Figures 5(c) and 5(d)). Furthermore, the effects of 10 *μ*M SalB were similar to those of ST2825, an inhibitor of MYD88, at a concentration of 10 *μ*M. These results are consistent with those observed *in vivo*.

### 3.6. Inflammation and Oxidative Stress Were Mediated In Vitro by Erk1/2/P38 via the TLR4/MyD88/NLRP3 Pathway

**Figure 6:**
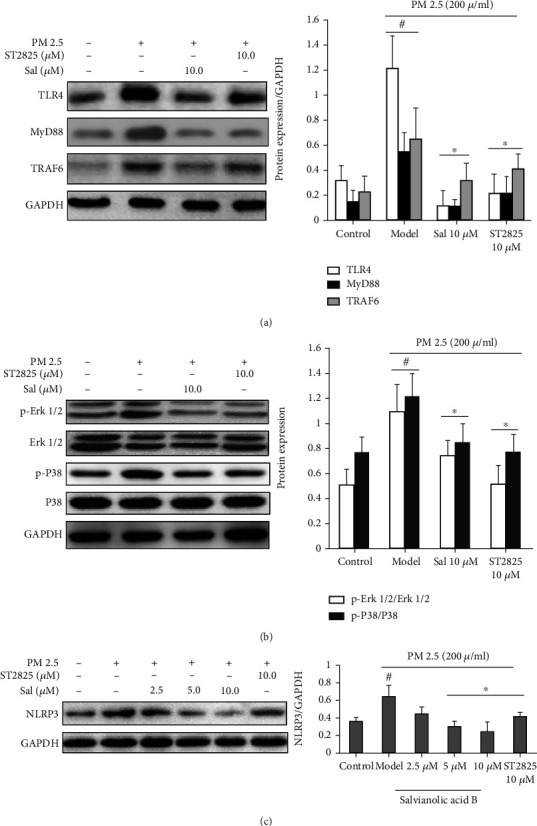
Inhibiting the expression of the TLR4/MyD88/TRAF-6 pathway suppressed the phosphorylation of their downstream signals (ERK1/2 and P38) in 16HBE cells. 16HBE cells were pretreated with 10 *μ*M SalB or ST2825 for 0.5 h prior to exposure to PM2.5 (200 *μ*g/mL) for 0.5 h. Cell lysates were then immunoprecipitated. (a) The expression levels of TLR4, MyD88, and TRAF-6. (b) The phosphorylation of ERK1/2 and P38. (c) Treatment with SalB reduced the expression of the NLRP3 inflammasome in a dose-dependent manner. Data represent mean ± S.E.M. from three independent experiments. ^#^*P* < 0.05 compared with the control group (PM2.5 untreated); ^∗^*P* < 0.01, compared with the model group (exposure to PM2.5).

As shown in Figure 6, both SalB and ST2825 significantly inhibited the expression of TLR4, MyD88, and TRAF-6, as well as phosphorylation of their downstream signals ERK1/2 and P38 (Figures 6(a) and 6(b)). Finally, we found that SalB reduced the expression of the NLRP3 inflammasome in a dose-dependent manner (Figure 6(c)). These results are consistent with those observed *in vivo*. These data suggest that the potential effects of SalB on PM2.5-induced airway inflammation and oxidative stress were mainly mediated via the TLR4/MyD88/NLRP signaling pathway.

## 4. Discussion

**Figure 7:**
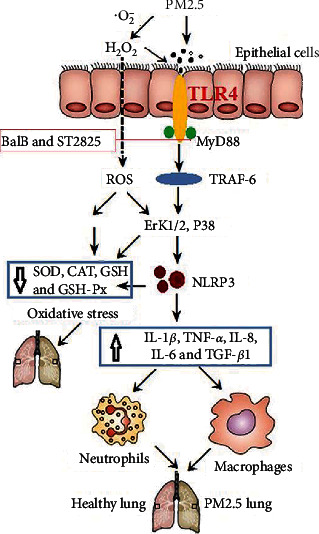
Scheme summarizing the protective effects of SalB inhalation on PM2.5-induced inflammatory and oxidative stress. SalB and ST2825 resulted in effective protection against PM2.5-induced acute airway inflammation and oxidative stress. These effects were closely correlated to the inhibition of the TLR4/MyD88/TRAF-6/NLRP3 pathway and the activation of the ERK1/2 and P38 signaling pathway.

In this study, we first showed that PM2.5-induced inflammatory cell infiltration in the lung tissue is related to the increased expression levels of MyD88 protein, thus suggesting that MyD88 may regulate PM2.5-induced acute airway inflammation. Inhalation of a MyD88 inhibitor (ST2825) and SalB significantly reduced PM2.5-induced acute airway inflammation and oxidative stress, reduced the release of inflammatory cytokines, and prevented the increase of ROS and the reduction of antioxidant factors in lung tissue and in human epithelial cells. Furthermore, inhalation of the MyD88 inhibitor and SalB also inhibited the TLR4/MyD88/TRAF-6/NLRP3 pathway, as well as the downstream pathway (ERK1/2 and P38) (Figure 7).

In China, there are many natural herbal medicines and traditional Chinese medicines that can be used to treat respiratory inflammation; these are usually safe and effective. Therefore, we are particularly interested in studying the effect of SalB on PM2.5-induced airway inflammation and oxidative stress. Air pollution (PM2.5) first affects the health of the respiratory tract. Since the respiratory tract is an open organ, air pollution and especially PM2.5 particles can readily damage the respiratory system by lowering the defenses of the airway epithelium and by altering immune responses. Over recent years, researchers have attempted to prevent and control the cellular and organ damage caused by PM2.5. Some researchers have shown that some natural compounds and traditional Chinese medicines can protect the cell and organ damage induced by PM2.5 both *in vitro* and *in vivo*, including calycosin [17], isovitexin [18], astaxanthin [19], curcumin [20], astragalus [21], codonopsis pilosula [21], ophiopogonin [22], triptolide [23], astragaloside IV [24], resveratrol [25], and lentinan [26]. All of these studies were carried out *in vivo*; drugs were administered systematically, by either oral or injection routes. In addition, Let-7a, a member of the microRNA family, also regulates PM2.5-induced oxidative stress and damage in human airway epithelial cells by targeting arginase 2 *in vitro* [27]. In the present study, we proved for the first time that the inhalation of SalB can significantly reduce the PM2.5-induced increase in the numbers of neutrophils and macrophages in BALF, decrease the infiltration of neutrophils and macrophages in lung tissue, and markedly decrease the levels of SOD, CAT, GSH, and GSH-Px. Treatment with SalB reduced the PM2.5-induced increase in the mRNA expression and protein cancel levels of IL-1*β*, TNF-*α*, KC, and TGF-*β*1 in mouse lung tissues cancel in a dose-dependent manner. Our results also showed that exposure to PM2.5 induced a significant increase in the mRNA expression and protein cancel levels of TLR4, MyD88, TRAF-6, and NLRP3 cancel in mouse lung tissues; these effects were reduced by SalB treatment in a dose-dependent manner. Next, we tried to verify the *in vivo* effect of SalB and found that PM2.5 stimulated an increase in the release of inflammatory cytokines and a reduction in the levels of antioxidant factors in human bronchial epithelial (16HBE) cells. SalB was also shown to inhibit the release of IL-1*β*, TNF-*α*, IL-8, and TGF-*β*1 in a dose-dependent manner and prevent the reduction of SOD, CAT, GSH, and GSH-Px levels. Consistent with our *in vivo* results, SalB also significantly inhibited the expression levels of TLR4, MyD88, and TRAF-6 proteins. Further *in vitro* studies on the downstream signaling pathways (TLR4/MyD88/TRAF-6) showed that SalB could significantly reduce the phosphorylation of ERK1/2 and P38. An inhibitor of MyD88 (ST2825) was used consistently as a control drug for all *in vivo* and *in vitro* experiments. Previous studies have shown that PM2.5 promotes plaque vulnerability at different stages of atherosclerosis, the formation of foam cells [8], and lung injury [4] and exacerbates allergic inflammation in the murine lung [4, 28] *via* the TLR4/MyD88/NF*κ*B pathway. Previous research, involving MyD88^−/−^ mice, showed that the exacerbation of PM2.5-enhanced ovalbumin allergic inflammation was significantly inhibited [28]. Furthermore, PM2.5-exposure has been shown to trigger increased expression of proinflammatory mediators (IL-6, IL-12, and MCP-1) and attenuate the influx of neutrophils into the pulmonary alveoli [29]. In addition, the MyD88 inhibitor LM9 has been shown to prevent the pathogenesis of atherosclerosis, accompanied by reduced vascular inflammation and oxidative stress in ApoE^−/−^ mice fed upon a high-fat diet [30]. These previous studies highlighted the translational role of MyD88 as a potential therapeutic target for the treatment of PM2.5-triggered airway inflammation and oxidative stress. In the present study, we proved, for the first time, that the mechanisms underlying the action of SalB are similar to those underlying the effects of the MyD88 inhibitor ST2825, thus suggesting that SalB is a natural compound that can act in the same way as the MyD88 inhibitor; the detailed action mechanism of SalB will be further studied by us.

## 5. Conclusion

Here, we demonstrate that the inhibition of the TLR4/MyD88/NLRP3 pathway is an effective means of treating inflammation and oxidative stress. Treatment with a MyD88 inhibitor (ST2825) and SalB significantly prevented and controlled PM2.5-triggered airway inflammation and oxidative stress. The inhalation of SalB allowed direct targeting of the drug directly to the target organs, thus improving its bioavailability, enhancing its efficacy, and reducing adverse systemic effects during the treatment of PM2.5-induced acute airway inflammation.

## Figures and Tables

**Table 1:** Primers for quantitative real-time PCR analysis.

Gene	Primer sequence (5′-3′)	Tm (°C)
Mouse IL-1*β*	Sense CGAGGCAGTATCACTCATTG	58
	Antisense CGTTGCTTGGTTC- TCCTTGT	

Mouse TNF-*α*	Sense CAGACCCTCACACTCAGATCATCTT	58
	Antisense TCGTAGCAAACCACCAAGTGG	

Mouse KC	Sense GCTGGGATTCACCTCAAGAA	58
	Antisense TGGGGACAAATTTTAGCATC	

Mouse TGF-*β*_1_	Sense TGAGTGGCTGTCTTTTGACG	58
	Antisense TCTCTGTGGAGCTGAAGCAA	

Mouse TLR4	Sense CTCACAACTTCAGTGGCTGGATTTA	58
	Antisense GTCTCCACAGCCACCAGATTCTC	

Mouse MyD88	Sense ATACCAACCCTTGCACCAAGTC	58
	Antisense TCAGGCTCCAAGTCAGCTCATC	

Mouse TRAF-6-	Sense TTTGGCGTCGGAGACACTTG	58
	Antisense TCGCTTGAAGACTGGCTGGA	

Mouse NLRP3	Sense AGATTACCCGCCCGAGAAAG	58
	Antisense TCCCAGCAAACCCATCCACT	

Mouse GAPDH	Sense AACTTTGGCATTGTGGAAGG	58
	Antisense AACTTTGGCATTGTGGAAGG	

## Data Availability

The analyzed datasets generated during the study are available from the corresponding author on reasonable request.

## Abbreviations

TLR4: Toll-like receptor 4
MyD88: Myeloid differentiation primary response 88
TRAF-6: TNF receptor associated factor 6
NLRP3: NOD-like receptor protein 3
Elisa: Enzyme-linked immunosorbent assays
RT-PCR: Real-time quantitative polymerase chain reaction
IL: Interleukin
TNF: Tumor necrosis factor
KC: Keratinocyte
TGF: Transforming growth factor
SOD: Superoxide dismutase
CAT: Catalase
GSH: Glutathione
GSH-Px: Glutathione peroxidase
ROS: Reactive oxygen species
Erk1/2: Extracellular signal regulated kinase 1/2
P38: P38 MAP kinase
DCFH-DA: 2′,7′-Dichlorodihydrofluorescein diacetate
HPLC: High-performance liquid chromatography
FBS: Fetal bovine serum
BALF: Bronchoalveolar lavage fluid
PBS: Phosphate buffer solution
BSA: Bovine serum albumin
H&E: Hematoxylin-eosin
IHC: Immunohistochemical
RIPA: Radioimmunoprecipitation
SDS-PAGE: Sodium dodecyl sulfate-polyacrylamide gel electrophoresis
PVDF: Polyvinylidene fluoride.

## Supplementary Materials

Supplementary Materials Comparison of SalB concentrations in blood and lung tissue between aerosol inhalation and intragastric administration in mice. Supplementary Table S1: lung tissue and blood concentrations after inhalation and intragastric administration. Figure S1: drug concentrations in blood and lung tissues of mice. Figure S2: standard curves applicable to the detection range of lung tissue samples and preparation of quality control samples. (a) SalB ion scan; (b) internal standard ion scan; (c) blank plasma chromatogram of rat; (d) chromatogram of rat plasma sample; (e) chromatogram of blank lung tissue in mice; (f) chromatogram of mouse lung tissue samples.
