# Supplementary material for: Inhalation of Salvianolic Acid B Prevents Fine Particulate Matter-Induced Acute Airway Inflammation and Oxidative Stress by Downregulating the LTR4/MyD88/NLRP3 Pathway
Source: Oxid Med Cell Longev. 2022 Jun 27;2022:5044356. doi: 10.1155/2022/5044356 (PMC9252752; doi:10.1155/2022/5044356)
Supplement: Supplementary Materials — Comparison of SalB concentrations in blood and lung tissue between aerosol inhalation and intragastric administration in mice. Supplementary Table S1: lung tissue and blood concentrations after inhalation and intragastric administration. Figure S1: drug concentrations in blood and lung tissues of mice. Figure S2: standard curves applicable to the detection range of lung tissue samples and preparation of quality control samples. (a) SalB ion scan; (b) internal standard ion scan; (c) blank plasma chromatogram of rat; (d) chromatogram of rat plasma sample; (e) chromatogram of blank lung tissue in mice; (f) chromatogram of mouse lung tissue samples. [file 5044356.f1.docx]

**Supplementary data 1**

**Comparison of SalB concentrations in blood and lung tissue between aerosol inhalation and intragastric administration in mice**.

**1. Grouping and administration:** Mice were randomly divided into 4 groups with 4 males and 4 females in each group, including single inhalation (7.5 mg/kg) group, single gavage (40 mg/kg) group, multiple inhalation (7.5 mg/kg) group and multiple gavage (40 mg/kg) group, respectively. Multiple administration group, once a day for 5 consecutive days.

**2. Sampling method:**

**2.1 Blood collection:** 1 h after the end of drug administration, the abdominal aorta blood was taken and placed in an EDTA-K2 anticoagulant tube, centrifuged at 5000 RPM for 10 min, and the upper plasma was separated and stored in a refrigerator at -80℃ for testing.

**2.2 Lung tissue sampling:** After blood was collected, the lung tissue was extracted quickly, weighed at the same location, and appropriate amount of normal saline was added. Homogenate of the tissue mass to the volume ratio of normal saline was 1:4 (g:mL), and stored in the refrigerator at -80℃ for testing by centrifugation.

**2.3 The sample testing:** The concentration of SalB in mice plasma and lung tissue was determined by HPLC-MS /MS.

**2.4 Data entry and statistical analysis:** Excel input and SPSS (Version 20) software package statistical processing, expressed as mean ± standard error. All data were tested for homogeneity of variance. If the variance is uniform (P<0.05), one-way ANOVA was performed, and Dunnet test was performed among each group. If variance was not uniform (P≤0.05), nonparametric test was performed, and Mann-Whitney U test was performed among groups. P<0.05 was statistically different. In comparison between the two groups, independent sample T test was performed and P<0.05 was considered statistically different.

**3. Results**

**3.1 Blood drug concentration:** The results showed that the blood drug concentration was 5.99 ± 2.30 (ng/mL) in single inhalation (7.5 mg/kg, n =8) group, and 14.15 ± 3.94 (ng/mL) in single gavage group (40 mg/kg, n=8) group at 1 h after administration. After 5 consecutive administration, the plasma concentration at 1 h after the end of administration was 7.94 ± 6.59 (ng/mL) in multiple inhalation group (7.5 mg/kg, n =8), and 38.81 ± 15.11 (ng/mL) in multiple gavage group (40 mg/kg, n =8).

**3.2 Drug concentration in lung tissue:** The results showed that the lung tissue concentration was 4826.25 ± 801.11 (ng/g) in single aerosol inhalation (7.5 mg/kg) group and 25.17 ± 2.93 (ng/g) in single gavage group (40 mg/kg) at 1 h after administration. Single inhalation group dose was less than 5.3 folds of single gavage group, while lung tissue concentration was about 192 folds of multiple gavage group (40 mg/kg) (P<0.001); After 5 consecutive administration, the lung tissue concentration was 21018.75 ± 2625.65 (ng/g) in multiple inhalation group (7.5 mg/kg), and 185.64 ± 55.88 (ng/g) in multiple gavage group (40 mg/kg). It was 113 times higher than that in multiple gavage group (40 mg/kg) (P<0.01).

The above results suggest that the drug concentration by inhalation administration maintaining lung tissue (target organ) is much higher than that of gavage administration when the dose of inhalation is lower than that of gavage administration.

**Table S1. Lung tissue and blood concentrations after inhalation and intragastric administration（‾x ± SEM）**

| Groups | Lung tissues（ng/g） | Plasma（ng/mL） |
| --- | --- | --- |
| single inhalation（7.5 mg/kg） | 4826.25±801.11 | 5.99±2.30 |
| single gavage（40 mg/kg） | 25.17±2.93^###^ | 14.15±0.94 |
| multiple inhalation（7.5 mg/kg） | 21018.75±2625.65^###^ | 7.94±6.59 |
| multiple gavage（40 mg/kg） | 185.64±55.88^**^ | 38.81±15.11 |

‾x ± SEM, n=8, one way ANOVA, comparison with single inhalation group, ^###^ P<0.001; comparison with multiple inhalation group, **P<0.01.


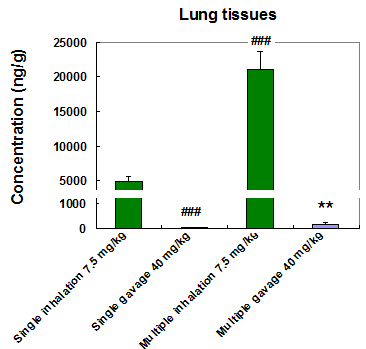


**Figure S1 Drug concentrations in blood and lung tissues of mice.** ‾x ± SEM, n=8, one way ANOVA, inhalation comparison with single inhalation group, ^###^ P<0.001; multiple gavage group compared with multiple inhalation group, **P<0.01.

**Biological sample analysis of SalB concentration in plasma and lung tissue of mice after inhalation and intragastric administration of SalB**

**The sample testing methods**

1. Objective : The purpose of this study was to determine the concentration of SalB in mouse plasma and lung tissue using liquid mass spectrometry (HPLC-MS/MS)

2. Analytical Methods: The concentration of SalB in mouse plasma and lung tissue was determined by liquid mass spectrometry (HPLC-MS/MS). The quantitative range of SalB in mouse plasma was 0.400-200 ng/mL, and that in mouse lung tissue was 2.00-1000 ng/mL.

2.1 Blank matrix: The blank matrix used in the detection of biological samples were SD mouse plasma (anticoagulant EDTA-K2) and mouse lung tissue (homogenate was normal saline). The blank matrix was provided by Hangzhou Brexmore Biomedical Technology Co., LTD., stored in refrigerator -20, and melt with water bath when in use.

2.2 Reagent

Acetonitrile: chromatographic pure, Merck Germany

Methanol: chromatographic pure, Merck Germany

Formic acid :HPLC grade, ACS

Methyl tert-butyl ether (MTBE): chromatographic pure, Merck Germany Ultra-pure water: self-made (Pure water meter model and manufacturer: Milli-Q, Millipore).

2.3 Main instrument

Analysis balance: Sartorius MCM36 Quality Comparator (Sartorius, Germany)

centrifuge: Beckman Coulter Allegra 64R

High Speed Freezing Centrifuge (Beckman Corporation, USA)

VORTEX mixer: Vortex-5 VORTEX oscillator (Its Linbell Instrument Manufacturing Company, Jiangsu Haimen, China)

Oscillator :TARGINTM VX-

Ultrasonic cleaner: KUDOS Ultrasonic Cleaner (Kedao Ultrasonic Instrument Co., LTD., Shanghai, China)

Pure water meter :Milli-Q ultra pure water system (Millipore Company, USA)

Pipette :(Eppendorf, Germany)

HPLC column :Waters Xbridge C8(4.6 100 mm,3.5 μm) Luna 5μm Phenyl-Hexyl 100A(2.0 50 mm,5 μm)

High Performance Liquid chromatography pump :LC-20AD binary pump,Shimadzu Company

Automatic sampler :SIL-20AC,Shimadzu

Column Temperature Chamber :CTO-20AC,Shimadzu

Mass Spectrometer :API 4000,AB Sciex

2.4 Chromatographic conditions

(1) Chromatographic conditions suitable for the detection of plasma samples (omitted)

(2) Chromatographic conditions suitable for the detection of lung tissue samples (omitted)

2.5 Mass spectrometry Condition

Ion source :Electrospray Ionization(ESI)

Ionization mode: Positive Ionization

mode: MRM Electrospray voltage (Ion)

Spray Voltage):5500 V

Turbo Ion Spray Temperature :550

Curtain Gas :20

Collision Gas (CAD):7

Nebulizing Gas (Gas1):60

Auxiliary Gas (Gas2):60

Inlet voltage (EP):10 Volts

Acquisition Time :3.2min

| Compound code | Compound | MRM | Standing time  （Time）（msec） | declustering potential  （DP）  （volts） | Collision energy  （CE）  （volts） | Impact chamber outlet voltage  （CXP）（volts） |
| --- | --- | --- | --- | --- | --- | --- |
| determinand | SalB | 705.3→392.4 | 100 | 130 | 50 | 12 |
| internal standard | chloromycetin | 383.0→337.5 | 100 | 100 | 30 | 10 |

2.6 Sample pretreatment process

(1) Pretreatment process suitable for solution sample detection

The calibrate standard solution quality control sample solution and solution sample are directly diluted uniformly, and mixed evenly with the internal standard solution of the same concentration before sample injection analysis.

(2) It is suitable for the pretreatment process of plasma sample detection.

(2.1) Take out frozen (or prepare fresh) calibrate standard sample and quality control sample blank matrix and test sample to thaw under wet ice;

(2.2) Swirl all samples evenly;

(2.3) Take 50.0μL calibrate standard quality control sample double blank sample zero control quality control sample Test sample into 1.5 mL EP tube, reagent blank take 50.0μL water;

(2.4) Return the test sample to the freezer;

(2.5) Using a continuous pipette, add 20.0μL of internal standard I02 to the sample. For double blank sample and reagent blank sample, add 20.0μL R01, and swirl for at least 1min;

(2.6) Using a continuous pipette, 50.0μL of 20mM ammonium acetate aqueous solution was added to all samples, and the mixture was oscillated for at least 5min;

(2.7) Using a continuous pipette, add 800μLMTBE to all samples, vortex oscillation mixing for at least 5min, 4℃ 10000rpm centrifugation for 5min;

(2.8) Take 700μL supernatant into a clean 2.0 mL 96-well plate and blow dry it at 4℃ in a nitrogen blower. Add 150μL complex solution (methanol: water (80:20)) to all samples for redissolution;

(2.9) Cover the plastic mat with 96-well plate, swirl and shake the mixture for at least 5min, then transfer it to automatic sampler or 4 ℃ refrigerator for storage until sampling.

(3) Suitable for the pretreatment process of lung tissue sample detection

(3.1) Take out frozen (or prepare fresh) calibrate standard sample and quality control sample blank matrix and test sample to thaw under wet ice;

(3.2) Swirl all samples evenly;

(3.3) take 40.0μL calibrate standard quality control sample double blank sample zero control quality control sample Test sample into 1.5 mL EP tube, reagent blank take 40.0μL water;

(3.4)Return the test sample to the frozen refrigerator;

(3.5) Add 100μL of 20mM ammonium acetate aqueous solution (containing 0.1% formic acid) to all samples using a continuous pipetor, and swirl for at least 5min;

(3.6) Add 400μL internal standard acetonitrile solution (internal standard concentration: 50ng/mL) in a continuous pipetor, swirl and mix for at least 5min, then centrifuge at 13000rpm for 10min at 4℃;

(3.7) Take 200μL supernatant into a clean 2.0ml 96-well plate, then add 200μL complex solution (methanol: water (80:20)) to all samples for redissolution;

(3.8)Cover the plastic mat with 96-well plate, swirl and shake the mixture for at least 5min, then transfer it to automatic sampler or 4℃ refrigerator for storage until sampling.

Sample storage tube Material: Polypropylene centrifugal tube

Internal standard name: chloromycetin

Sample pretreatment method: Plasma sample: liquid-liquid extraction (extraction agent :MTBE) Lung tissue sample: protein precipitation

Injection volume: Plasma sample :5.00 μL Lung tissue sample :10.00 μL

Standard curve regression method/weight: The mouseio of the chromatographic peak area of the tested substance to the corresponding internal standard was the vertical coordinate, and the linear regression was performed by weighting (W= L/X2) with the concentration (X) of the tested substance in plasma and the peak area mouseio (Y). The regression equation (Y=a+ Bx) obtained was the standard curve;

linearity range (ng/mL) : 0.400-200 ng/mL for plasma samples, Lung tissue sample: 2.00-1000 ng/mL

Quality control sample concentration (ng/mL) :Refer to 12 for details.

Standard curve and quantitative method of prepamouseion of quality control samples Chromatographic peak area mouseio between the measured and the internal standard was the comparison of the accuracy of weighing of the main reserve fluid in ordinate analysis: 3 (solution samples), 40 (mouse plasma samples), 40 (mouse lung tissue samples) ;

The difference between the two parallel weighing reserves (used to prepare calibrate standard sample and working solution of quality control sample respectively) was -0.2 %, and the result showed that the weighing was accumousee and reliable

Sample collection: Plasma samples and lung tissue samples of mouse were provided by our Lab.

4.1Preparation of standard curves and quality control samples

4.1.1Preparation of reserve solution (1) SalB reserve solution (0.200 mg/mL) [AS1] Accurately weigh SalB(about 1mg, and record the weighing weight), calculate the total correction factor (including water content, salt purity, etc.), calculate the actual weight of SalB based on the total correction factor, dissolve it in methanol, and obtain the final concentration of 0.200mg /mL

(2) Preparation of internal standard Chloromycetin reserve solution (0.200mg /mL) [IS1] accurately weighed Chloromycetin (approx. 1mg, recorded weight) and calculated the total correction factor (including water content salt) The actual weight of Chloromycetin was calculated according to the total correction factor. The final concentration of Chloromycetin was 0.200 mg/mL after it was dissolved in methanol.

4.1.2 Preparation of the working fluid of the object to be tested

(1) Preparation of the working fluid of the object to be tested is applicable to the testing range of solution samples;

(2) It is suitable for preparing the working fluid of the substance to be tested within the detection range of plasma samples;

(3) It is suitable for preparation of working fluid of the substance to be measured within the detection range of lung tissue samples.

4.1.3 Preparation of internal standard working fluid.

4.1.4 Preparation of standard curves and quality control samples.

(1) Standard curves applicable to the detection range of plasma samples and preparation of quality control samples;

(2) Standard curves applicable to the detection range of lung tissue samples and preparation of quality control samples.

Figure S2: SalB ion scan

Figure S3: Internal standard ion scan

Figure S4: Blank plasma chromatogram of mice

Figure S5: Chromatogram of mouse plasma sample

Figure S6: Chromatogram of blank lung tissue in mice

Figure S7: Chromatogram of mouse lung tissue samples
